# The efficacy and safety of tirofiban for patients with acute ischemic stroke: A protocol for systematic review and a meta-analysis

**DOI:** 10.1097/MD.0000000000014673

**Published:** 2019-03-01

**Authors:** Jiali Niu, Yunlong Ding, Tingting Zhai, Feng Ju, Tong Lu, Ting Xue, Dengyang Yin, Dong Fang, Hongjun Chen, Guangyu Zhao

**Affiliations:** a Department of Clinical Pharmacy; b Department of Neurology; c Department of Pharmacy, Jingjiang People's Hospital, the Seventh Affiliated Hospital of Yangzhou University, Jiangsu, China.

**Keywords:** acute ischemic stroke, meta-analysis, systematic review, tirofiban

## Abstract

**Background:**

The clinical use of tirofiban remains controversial for patients with acute ischemic stroke (AIS), we aimed to conduct a meta- analysis of cohort studies to assess the efficacy and safety of tirofiban for AIS patients.

**Methods:**

All apparently unconfounded randomized controlled trials (RCTs) and case-controlled studies, with or without blinding, of tirofiban in individuals with AIS will be included in this review. We will conduct a literature search in 2 databases Pubmed and Embase, using indexing terms related to cerebral infarctions and tirofiban to include articles indexed as of Jan 31, 2019 in the English language only. Two reviewers will independently select trials for inclusion and assess trial quality. Two pairs of review authors will independently extract information for each included trials. Primary outcomes are any intracerebral hemorrhage (aICH), symptomatic intracranial hemorrhage (sICH), fatal ICH, recanalization rate and long-term outcome. The risk of bias of the included studies will be evaluated based on Cochrane assessment tool. Revman 5.3 will be used for heterogeneity assessment, generating funnel-plots, data synthesis, subgroup analysis, and sensitivity analysis.

**Result:**

We will provide practical and targeted results assessing the efficacy and safety of tirofiban for AIS patients, to provide reference for clinical use of tirofiban.

**Conclusion:**

The stronger evidence about the efficacy and safety of tirofiban for AIS patients will be provided for clinicians.

## 1 Introduction

Antiplatelet therapy is one of the important treatments for acute ischemic stroke (AIS).^[1]^ Aspirin and clopidogrel are the most commonly used oral antiplatelet drugs, but they work slowly. In contrast with them, intravenous tirofiban is a fast-acting non-peptide glycoprotein IIb/IIIa (GPIIb/IIIa) receptor antagonist located on thrombocytes and with a short half-life that reverses bleeding time to normal within 4 hours after discontinuation.^[2]^

To date, the efficacy and safety of tirofiban has been investigated in several studies of acute stroke patients with controversial results. Promising data from the SaTIS^[3]^ and SETIS^[4]^ trials showed that tirofiban was safe and potential efficacy in acute stroke patients, whereas Kellert et al revealed negative results for tirofiban in acute stroke.^[5,6]^ A cohort study of emergent carotid artery stenting (eCAS) patients found neither a clear benefit nor increased risk for treatment compared tirofiban with aspirin.^[7]^ Since there is no agreement on whether tirofiban can be applied to AIS, further evidence is still needed for clinical practice. Therefore, we aim to conduct a meta- analysis of cohort studies to assess the efficacy and safety of tirofiban for AIS patients.

## 2 Methods

### 2.1 Registration

This systematic review protocol has been registered on PROSPERO as CRD42018110866. In this paper, the protocol will be performed according to the Cochrane Handbook for Systematic Reviews of Intervention.^[8]^ If we will refine procedures described in this protocol, we will document the amendments in the PROSPERO database and disclose them in future publications related to this meta-analysis.

### 2.2 Inclusion criteria for considering studies

#### 2.2.1 Types of studies

All apparently unconfounded randomized controlled trials (RCTs) and case-controlled studies, with or without blinding, of tirofiban in individuals with AIS will be included in this review.

#### 2.2.2 Types of participants

According to the World Health Organization's definition^[9]^ of AIS, all definite AIS patients regardless of race, region, sex will be included. All the participants must be made a definite diagnosis of AIS by brain computed tomography or magnetic resonance imaging.

#### 2.2.3 Types of interventions

Tirofiban administered early after an ischemic stroke via either intravenous or arterial.

#### 2.2.4 Types of outcome assessments

Any available information about safety and efficacy in both tirofiban and control groups will be assessed. Primary outcomes are any intracerebral hemorrhage (aICH), symptomatic intracranial hemorrhage (sICH), fatal ICH, in hospital mortality, recanalization rate and long-term outcome at least 3 months of follow-up. Recanalization is graded by the Thrombolysis in Cerebral Infarction (TICI), and the recanalization rate is defined as TICI score 2b or 3.

### 2.3 Search strategy

We will conduct a comprehensive literature search in 2 databases Pubmed and Embase, to include articles indexed as of Jan 31, 2019 in the English language only. The key search terms will be used are [(“cerebral infarctions” OR “infarctions, cerebral” OR “infarction, cerebral” OR “cerebral infarction, left hemisphere” OR “left hemisphere, infarction, cerebral” OR “infarction, right hemisphere, cerebral” OR “subcortical infarctions” OR “posterior choroidal artery infarction” OR “anterior choroidal artery infarction”) OR “brain infarctions” OR “infarction, brain” OR “infarctions, brain” OR “anterior circulation brain infarction” OR “infarction, brain, anterior circulation” OR “venous infarction, brain” OR “brain venous infarction” OR “brain infarction, posterior circulation” OR “posterior circulation brain infarction” OR “strokes” OR “cerebrovascular accident” OR “apoplexy, cerebrovascular” OR “cerebrovascular accident, acute” OR “acute strokes” OR “acute cerebrovascular accidents” AND (“N- (butylsulfonyl) -O- (4- (4-piperidyl) butyl) l tyrosine” OR “MK 383” OR “tirofiban hydrochloride” OR “aggrastat” OR “aggrastat” OR “tirofiban hydrochloride monohydrate” OR “L 700462) OR L-700462” OR “L-700,462” OR “tirofiban”]

### 2.4 Data collection

#### 2.4.1 Selection of studies

**Figure 1:**
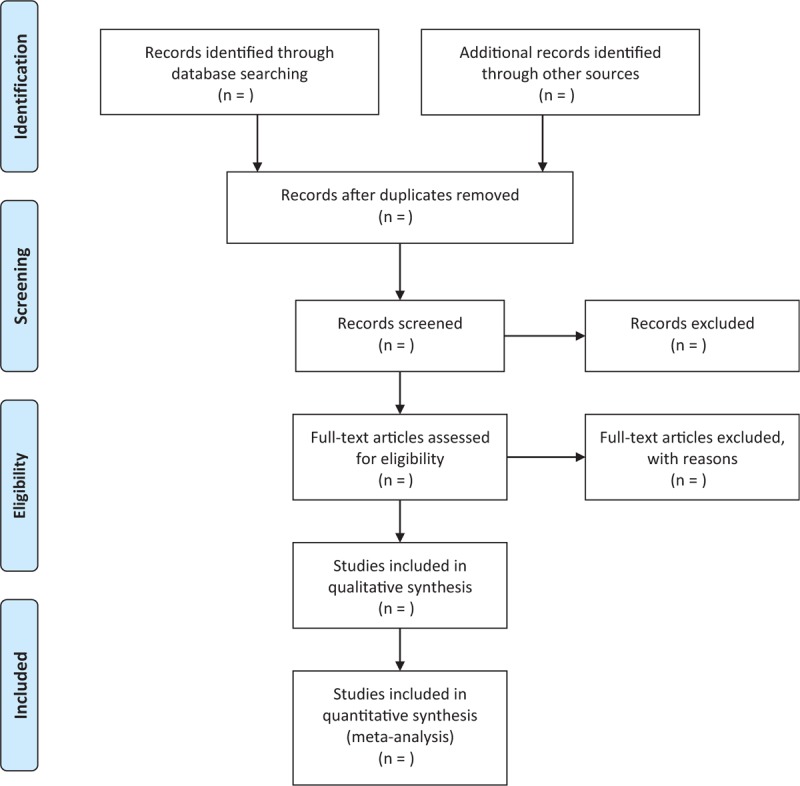
Flow diagram of the study selection process. *From:* Moher D, Liberati A, Tetzlaff J, Altman DG, The PRISMA Group (2009). Preferred Reporting Items for Systematic Reviews and Meta-Analyses: The PRISMA Statement. PLoS Med 6(6): e1000097. doi:10.1371/journal.pmed1000097.

Two reviewers will independently select trials for inclusion. All apparently unconfounded RCTs and case-controlled studies evaluated the efficacy and safety of tirofiban on AIS patients will be included. We will exclude articals if they meet any of the following criteria: (1) fewer than 10 patients; (2) studies not comparing tirofiban and none tirofiban, or tirofiban and conventional antiplatelet agents (aspirin and/ or clopidogrel). The specific process of study selection is shown in Figure 1.

#### 2.4.2 Data and information extraction

Two pairs of review authors will independently extract general information for each included trial, including the name of first author, year, country, design, sample size, average age, sex ratio, time from symptom onset, main treatment (thrombectomy/ alteplase), and the method for administration of tirofiban. The fifth author will check all the data.

In the same manner, we will extract data for efficacy and safety assessments. For each study, we will extract the following information: recanalization rate, bleeding complications, 24-hour National Institutes of Health Stroke Scale (NIHSS), perioperative morbidity, long-term good neurologic outcome, and long-term morbidity. Bleeding complications will be including aICH, sICH, and fatal ICH. Long-term outcome will require at least 3 months of follow-up. Good outcome is defined as modified Rankin scale (mRS) 0 to 2 and excellent outcome as mRS 0 to 1.

### 2.5 Assessment of risk of bias

Based on Cochrane assessment tool for risk of bias, 2 pairs of review authors will independently extract information for each included trial about the trial quality, including random sequence generation, allocation concealment, blinding of participants and personnel, blinding of outcome assessment, incomplete outcome data, selective reporting, and other bias. The fifth author will check all the data. We will contact the authors of the studies if the above information was not available in the published reports. We will use this information to evaluate quality and resolve disagreements by discussion until consensus is reached.

### 2.6 Data analysis

#### 2.6.1 Assessment of heterogeneity

We will use the chi-square test and *I*^2^ statistic to assess heterogeneity. It indicates that the heterogeneity exceeds the acceptable range when *P* < .10 or *I*^2^ > 50%. If the heterogeneity is in the acceptable range (*P* > .10, *I*^2^ < 50%), the fixed effect model shall be used for data analysis; otherwise, the random effect model will be adopted.

#### 2.6.2 Date synthesis

Meta-analysis and synthesis will be performed with RevMan 5.3 software provided by Cochrane collaboration. Risk ratio (OR) and 95% confifidence interval (95% CI) will be used for dichotomous variables, *P* values less than .05 will be considered statistically significant.

#### 2.6.3 Subgroup analysis

We will emplore the following subgroup analysis to explore the possible causes of high heterogeneity:

1. trials with low and high risk of bias;
2. articles with different impact factors (≥5, 3∼5, and ≤3);
3. patients received endovascular thrombectomy and not.

#### 2.6.4 Sensitivity analysis

We will also conduct sensitivity analysis by excluding merged trails one by one and observe whether the synthesis result changes significantly. If the changes significantly, we will reassess it to decide whether to merge it, and make a decision cautiously. If there is no significant changes, it indicates that our synthesized result is firm.

### 2.7 Assessment of publication bias

If more than 10 articles are available for quantitative analysis, we will generate funnel plots to assess publication bias. A symmetrical distribution of funnel plot data indicates that there is no publication bias, otherwise, we will analyze the possible cause and give reasonable interpretation for asymmetric funnel plots.

### 2.8 Confidence in cumulative evidence

GRADE system will be used for assessing the quality of our evidence.^[10]^ According to the grading system, the level of evidence will be rated high, moderate, low and very low.

## 3 Discussion

Stroke is one of the leading causes of death and disability all over the world.^[11]^ Antiplatelet therapy is an important treatment for ischemic stroke.^[12]^ Compared with the most commonly used oral antiplatelet drug clopidogrel and aspirin, intravenous tirofiban is fast-acting and is able to be used more quickly and effectively for patients with dysphagia.^[4]^ However, the application of tirofiban in ischemic stroke is a controversial issue.

In 2005, Mangiafico reported that the combination of tirofiban with intra-arterial urokinase and mechanical thrombolysis might improve the recanalization rate and result in a good outcome in patients with major cerebral arteries occlusions.^[13]^ Kwon considered it was feasible for intra-arterial tirofiban as an adjuvant after unsuccessful recanalization with urokinase for AIS.^[14]^ Kim reported that administration of local intra-arterial tirofiban after anterograde flow formation was a viable treatment strategy for reducing the risk of reocclusion after intraarterial thrombolysis for patients of AIS.^[15]^ Li suggested that it was safe and more effective to intravenous tirofiban immediately after alteplase compared with alteplase alone for selected stroke patients.^[16]^ However, some other scholars found negative^[7]^ or even opposite results.^[5,6]^

This study will conduct a meta-analysis of related RCTs and cohort studies, and provide the current evidence on the efficacy and safety of tirofiban for AIS, so as to better guide clinical practice.

## Author contributions

Jiali Niu, Yunlong Ding, Tingting Zhai, Guangyu Zhao and Hongjun Chen designed the systematic review. The protocol was drafted by Jiali Niu, Yunlong Ding, and revised by Hongjun Chen and Guangyu Zhao. Jiali Niu, Yunlong Ding developed the search strategy. Jiali Niu, Yunlong Ding, Tingting Zhai, Feng Ju, Tong Lu, Ting Xue, Dengyang Yin, Dong Fang will independently work on study selection, quality assessment, data extraction, and synthesis.

**Conceptualization:** Jiali Niu, Yunlong Ding, Guangyu Zhao.

**Data curation:** Jiali Niu, Yunlong Ding, Tingting Zhai, Dengyang Yin.

**Formal analysis:** Jiali Niu, Feng Ju, Tong Lu, Ting Xue, Dengyang Yin.

**Funding acquisition:** Yunlong Ding.

**Investigation:** Jiali Niu, Hongjun Chen, Guangyu Zhao.

**Methodology:** Jiali Niu, Dong Fang.

**Software:** Jiali Niu, Yunlong Ding.

**Supervision:** Hongjun Chen, Guangyu Zhao.

**Writing – original draft:** Jiali Niu, Yunlong Ding.

**Writing – review & editing:** Hongjun Chen, Guangyu Zhao.

Guangyu Zhao orcid: 0000-0002-5480-4202.
